# Heart rate variability as an independent predictor for 8-year mortality among chronic hemodialysis patients

**DOI:** 10.1038/s41598-020-57792-3

**Published:** 2020-01-21

**Authors:** Yu-Ming Chang, Ya-Ting Huang, I-Ling Chen, Chuan-Lan Yang, Show-Chin Leu, Hung-Li Su, Jsun-Liang Kao, Shih-Ching Tsai, Rong-Na Jhen, Chih-Chung Shiao

**Affiliations:** 1 Division of Nephrology, Department of Internal Medicine, Camillian Saint Mary’s Hospital Luodong, No. 160, Zhongheng S. Rd., Luodong, Yilan, 26546 Taiwan ROC; 2 Department of Nursing, Camillian Saint Mary’s Hospital Luodong, No. 160, Zhongheng S. Rd., Luodong, Yilan, 26546 Taiwan ROC; 3 Saint Mary’s Junior College of Medicine, Nursing and Management. No. 100, Ln. 265, Sec. 2, Sanxing Rd., Sanxing Township, Yilan County, 266 Taiwan ROC

**Keywords:** Haemodialysis, Risk factors, Interventional cardiology, Outcomes research

## Abstract

The repeated measurements of heart rate variability (HRV) is more relevant than a single HRV measurement in predicting patient prognosis but is less addressed previously. This prospective study aimed to investigate the association between repeated measurements of HRV and long-term mortality in chronic hemodialysis patients. The 164 patients (65.0 ± 13.1 years; woman, 57.3%) were enrolled from June 1, 2010, to August 31, 2010, and received four HRV measurements (before and during the index hemodialysis session) after the enrollment. The baseline characteristic and clinical variables, including mortality, were documented. The joint modeling method and Cox regression were used for statistical analyses. After an 8-year follow-up, 79 patients expired, and 85 patients survived. We found that higher normalized high-frequency (nHF) (hazard ratio [HR] 1.033) as well as lower very-low-frequency (HR 0.990), Variance (HR 0.991), normalized low-frequency (HR 0.999, P = 0.006), and low-frequency/high-frequency ratio (HR 0.796) were independent predictors for cardiovascular mortality. Whereas the independent predictors for infection-associated mortality included higher nHF (HR 1.033) as well as higher age (HR 19.29) and lower serum albumin (HR 0.01, P = 0.001). (all P < 0.001 unless otherwise stated) In conclusion, HRV measurement predicts long-term mortality among hemodialysis patients.

## Introduction

Autonomic nervous system (ANS) dysfunction is noted in more than 50 percent of chronic hemodialysis patients^1^. Among these patients, the mechanism of autonomic neuropathy is attributed to the reduced end-organ response of circulating catecholamines, disturbances in cardiac function, and derangements in the sympathetic and parasympathetic nervous system^2^. ANS dysfunction leads to hypertension, hypotension, and cardiac death in hemodialysis patients^3–5^. Heart rate variability (HRV), which represents the variation of the beat-to-beat interval, is a simple and noninvasive method to evaluate ANS functions that influence cardiovascular systems. HRV assessments include time-domain analysis, e.g., the standard deviation of normal to normal interval (SDNN), or frequency domain analysis which includes several indices such as total power (TP), very low frequency (VLF), low-frequency (LF), high-frequency (HF), and ratio of LF/HF^6^. Among these parameters, VLF is affected by the thermoregulation of the vasomotor tone. LF and normalized LF (nLF) activity indicate a mixture of both the sympathetic and parasympathetic effects. HF and normalized HF (nHF) activity have been related to parasympathetic nervous activity, which represents the vagal-mediated modulation of heart rate. LF/HF ratio is an index of sympathetic to parasympathetic balance. TP can be considered as the all spectra of the frequencies, whereas the variance of the R-R interval values (Variance) indicates parasympathetic activity or total activities of ANS.

The application of HRV measurement was initially focused on the prediction of survival in patients with ischemic heart disease^7,8^, but was subsequently extended to many different populations, including chronic kidney disease (CKD) patients. In CKD patients not on dialysis, lower HRV was significantly linked to increased risk of cardiovascular events, development of end-stage renal disease (ESRD) and mortality^9,10^. Whereas in chronic hemodialysis patients, HRV measurement showed an independent prognostic value on all-cause mortality or cardiac death^11–15^. Different from other investigators who evaluated the associated between a single HRV measurement at baseline or a specific time points with outcomes, Chen *et al*. evaluated the association between the “change of HRV values measured before and after HD” and patients’ prognoses^15^. Chen *et al*. found that the “change of HRV between post-hemodialysis and pre-hemodialysis values” is a useful predictive marker for overall and cardiovascular mortality among patients receiving hemodialysis. Moreover, the “delta values of HRV” exhibited a better predictive power than the single HRV measurement before hemodialysis^15^.

The dynamic change of HRV indices, which represents the responses or “reserve” of the ANS following the stimulation generated during the hemodialysis, is probably a more relevant predictor than a single HRV measurement. In this point of view, the work of Chen *et al*.^15^ was constructive and worthy of more attention. However, the real dynamic changes of HRV during the hemodialysis process are likely to be a “reverse V-shape,” which denotes “increase at the initial stage but decreases at a later stage^16–18^. The HRV measurements at two time-points before and after hemodialysis could not demonstrate the real dynamic changes of HRV during the hemodialysis.

The current study aimed to prove the hypothesis that the repeated measurements of HRV indices during the hemodialysis is a reliable marker for predicting long-term patient prognoses among patients receiving maintenance hemodialysis.

## Materials and Methods

### Ethical consideration

The study design conformed to the ethical guidelines of the 1975 Declaration of Helsinki and was approved by the Institutional Review Board of Camillian Saint Mary’s Hospital Luodong (SMHIRB_105009). The study was performed following the study protocol and relevant guidelines. Written informed consent was obtained from all enrolled patients, and the data were analyzed anonymously.

### Study design and populations

This prospective study was carried out using a cohort of 175 stable hemodialysis patients, which was created in a teaching hospital during the period of June 1, 201, to August 31, 2010. The inclusion criteria of this cohort included adult patients (>18 years of age) who received chronic hemodialysis for at least three months. While the exclusion criteria contained patients who had an arrhythmia or active infection, and who did not agree to receive HRV measurement.

HRVs were measured using an analyzer (SSIC, Enjoy Research Inc., Taiwan). The enrolled participants received four HRV measurements at the time point before (HRV-0) and during the index hemodialysis (namely, HRV-1, -2, and -3 at the initial, middle, and late phases of the hemodialysis, respectively). The index hemodialysis was performed for four hours using dialysate with a temperature of 36.5 °C. HRVs were expressed as standard frequency-domain measurements, including VLF (0.003–0.04 Hz), LF (0.04–0.15 Hz), HF (0.15–0.40 Hz), TP, LF/HF ratio, Variance, nLF, and nHF^6,19^. The baseline characteristic information, including demographic data, comorbid diseases, laboratory tests, and medications, were obtained from patients’ medical charts at enrollment. The details of performing HRV measurement and obtaining patients’ information were clearly stated in our previous work^16,17^. After the enrollment and HRV measurements, these participants were continuously followed for eight years until August 2018.

### Outcomes

The endpoints of this study were all-cause mortality censored at eight years. The censoring period was calculated from the date of HRV measurements to the date of death for the non-survivors or eight years for the survivors. Then we further categorized the all-cause mortality into cardiovascular mortality and infection-associated mortality and evaluated the independent predictors of these two entities.

### Statistical analysis

Categorical variables were expressed as “numbers (percentages).” Continuous variables were expressed as “mean ± standard deviation (SD)” for normal distribution variables and “median (interquartile range [IQR])” for non-normal distribution variables.

The statistical analyses were performed using the Scientific Package for Social Science (PASW Statistics for Windows, Version 22.0, Chicago: SPSS Inc.) and R3.3.1 (R Foundation for Statistical Computing, Vienna, Austria). A two-sided P ≤ 0.05 was considered statistically significant in all statistical analyses, Chi-square test and independent t-test were used to compare categorical variables and continuous variables between two groups, respectively. The mixed model was used to compare the differences among the values of the four measurements (HRV-0 to -3) of the individual HRV indices and the beta coefficients (B) of the individual HRV indices. Further, the multivariate mixed model was applied to calculate the adjusted B of the individual HRV indices. In this step, all variables put into the mixed model needed to pass the collinearity test. The variable insignificant in the model would be deleted one after another until significance is shown in the mixed model. The first-order autoregression covariance model (AR1) was used to test the influence of HRV indices. Next step, multivariate Cox regression with the stepwise method was applied to determine the independent risk factors for mortality among baseline characteristics and procedures.

**Figure 1:**
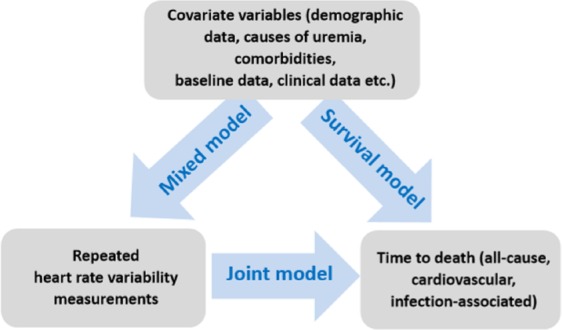
The diagrammatical representation of the joint model.

Finally, the joint modeling method was applied to determine the independent predictors among the HRV indices with adjustment to the independent risk factors found in the multivariate Cox regression. The joint modeling method could perform simultaneous analysis of repeated measurements and survival data, which was an impossible task traditionally^20^. The diagrammatical representation of the joint model for repeatedly measured data and survival data was depicted in Fig. 1.

The main objective of the current study is to build a joint model, to simultaneously modeling the repeated HRV measurements and time to death and to link them using unobserved random effects through the use of a shared parameter model. For the calculation of expected survival probabilities, the Monte Carlo scheme which uses random sampling and statistical modeling to estimate mathematical functions and mimic the operations of complex systems is implemented that accepts as main arguments a fitted joint model, and a data frame that contains the longitudinal and covariate information for the subjects for which we wish to calculate the predicted survival probabilities and plot the graph of survival probabilities related with HRV indices^20,21^.

## Results

During the 8-year follow-up, 11 patients were excluded due to loss of follow-up and missing data, whereas the rest 164 patients (mean age, 65.0 ± 13.1 years; woman, 57.3%) were enrolled into the final statistical analyses. Among the 164 participants, 79 (48.2%) patients died, whereas 85 (51.8%) patients kept alive during the 8-year follow-up period. Among the 79 patients who died during follow-up, 13 patients died from cardiovascular causes, and 49 died from infectious causes.

### Demographic data and HRV indices between the two groups

Compared with the survivors, the non-survivors were significantly older (69.2 ± 13.3 versus 61.2 ± 11.8, P < 0.001) and with higher cardiothoracic ratio (52.6 ± 5.4% versus 50.5 ± 4.4%, P = 0.006), but had lower baseline hemoglobin (9.4 ± 1.5 g/dL versus 10.0 ± 1.3 g/dL, P = 0.006), blood urea nitrogen (70.7 ± 19.6 mg/dL versus 79.4 ± 19.6 mg/dL, P = 0.005), phosphate (4.7 ± 1.7 mg/dL versus 5.2 ± 1.6 mg/dL, P = 0.048) and albumin (3.7 ± 0.3 g/dL versus 3.9 ± 0.3 g/dL, P = 0.002). Other demographic data, causes of uremia, comorbidities, baseline laboratory data, and the data at the index hemodialysis were not statistically different between the two groups (Table 1). Besides, all the relevant baseline and clinical variables passed the collinearity test (Table S1).

**Table 1:** Comparisons of the baseline characteristics and clinical variables between survivors and non-survivors at 8-year follow-up.

	Survivors (n = 85)	Non-survivors (n = 79)	p-value
Gender, woman	49 (57.6%)	45 (57.0%)	0.929
Age, years	61.2 ± 11.8	69.2 ± 13.3	<0.001
Period of dialysis, years	6.4 ± 5.9	5.1 ± 4.9	0.129
**Causes of uremia**			0.143
Diabetic nephropathy	22 (25.9%)	29 (36.7%)	
Hypertension	2 (2.4%)	0 (0.0%)	
Chronic glomerulonephritis	44 (51.8%)	42 (53.2%)	
Others	17 (20.0%)	8 (10.1%)	
**Comorbidities**			
Diabetes mellitus	27 (31.8%)	34 (43.0%)	0.136
Hypertension	58 (68.2%)	61 (77.2%)	0.198
Liver cirrhosis	5 (5.9%)	9 (11.4%)	0.207
Coronary artery disease	19 (22.4%)	20 (25.3%)	0.656
Heart failure	18 (21.2%)	22 (27.8%)	0.320
Cerebrovascular accident	8 (9.4%)	12 (15.2%)	0.259
Peripheral arterial disease	8 (9.4%)	4 (5.1%)	0.285
Chronic obstructive pulmonary disease	6 (7.1%)	11 (13.9%)	0.150
Malignancy	4 (4.7%)	9 (11.4%)	0.113
**Baseline data**			
Kt/V	1.4 ± 0.2	1.4 ± 0.2	0.991
Urea Reduction Ratio, %	82.1 ± 78.3	74.9 ± 8.0	0.413
Cardio-Thoracic Ratio, %	50.5 ± 4.4	52.6 ± 5.4	0.006
White blood cell, x10^9^/L	6.6 ± 2.3	6.0 ± 1.9	0.075
Hemoglobin, g/dL	10.0 ± 1.3	9.4 ± 1.5	0.006
Blood urea nitrogen, mg/dL	79.4 ± 19.6	70.7 ± 19.6	0.005
Creatinine, mg/dL	10.8 ± 2.3	10.5 ± 4.5	0.549
Calcium, mg/dL	9.0 ± 0.8	9.1 ± 0.6	0.886
Phosphate, mg/dL	5.2 ± 1.6	4.7 ± 1.7	0.048
Calcium phosphate product, (mg/dL)^2^	47.1 ± 14.9	42.9 ± 15.6	0.075
Albumin, g/dL	3.9 ± 0.3	3.7 ± 0.3	0.002
Sodium, mmol/L	138.0 ± 3.4	137.5 ± 3.1	0.355
Potassium, mEq/L	4.7 ± 0.7	4.7 ± 0.9	0.901
Intact-parathyroid hormone, ug/L	345.6 ± 615.1	223.1 ± 287.8	0.101
Total cholesterol, mg/dL	159.0 ± 31.5	167.7 ± 40.4	0.129
Triglyceride, mg/dL	141.4 ± 118.8	174.9 ± 129.6	0.086
Low-density lipoprotein, mg/dL	98.8 ± 28.5	99.2 ± 32.8	0.941
High-density lipoprotein, mg/dL	34.8 ± 15.3	34.8 ± 20.0	0.988
Sugar (non-fasting), mg/dL	150.2 ± 55.7	145.3 ± 55.3	0.571
nPCR, g/kg/day	1.1 ± 0.4	1.1 ± 0.4	0.413
Body mass index, kg/m^2^	21.8 ± 3.6	22.4 ± 4.2	0.344
**Data at the index hemodialysis**			
Dry weight, kg	55.3 ± 11.4	55.6 ± 10.5	0.873
Actual ultrafiltration, kg	2.2 ± 1.0	2.3 ± 0.9	0.679
%Ultrafiltration, %	4.0 ± 1.7	4.1 ± 1.6	0.535
MAP at initial of hemodialysis, mmHg	92.0 ± 15.3	90.6 ± 14.7	0.547

Notes: Values are presented as the mean ± standard deviation for continuous variables or number (%) for categorical variables unless otherwise stated. P-value was calculated using a Chi-square test or independent student’s *t*-test as appropriate. Baseline laboratory data were the pre-dialysis data obtained when patients are receiving HRV measurements.

Abbreviations: i-PTH, intact-parathyroid hormone; MAP, mean arterial pressure; nPCR, normalized protein catabolic rate.

Besides, none of the individual HRV indices at any of the four time-points were of significant difference between survivors and non-survivors (Table 2). The comparisons of HRV indices between survivors and non-survivors after an 8-year follow-up period were exhibited in Fig. 1. Roughly speaking, most of the HRV indices (except nHF) tended to increase initially (HRV-0 to HRV-2), but the tendency of elevation or even the values decreased in the later phase (HRV-2 to HRV-3) of hemodialysis in both groups. The tendency of “decreasing values” in the later phase was more significant in non-survivors than in survivors. Moreover, these HRV indices were generally higher in survivors than non-survivors at most of the four time-points.

**Table 2:** Comparisons of the heart rate variability indices between survivors and non-survivors at 8-year follow-up.

	Survivors (n = 85)	Non-survivors (n = 79)	p-value
**Before the index hemodialysis session**^**+**^			
VLF-0	4.60 (3.77–5.51)	4.14 (3.07–5.68)	0.269
TP-0	5.28 (4.26–6.28)	5.21 (4.12–6.37)	0.813
Variance-0	5.54 (4.61–6.35)	5.27 (4.16–6.55)	0.676
nLF-0	41.40 (28.63–62.70)	37.60 (19.30–51.40)	0.177
nHF-0	31.55 (19.53–45.08)	31.40 (21.05–38.43)	0.417
LF/HF-0	0.20 (0.01–1.20)	0.05 (0.01–0.81)	0.472
**Initial phase of the index hemodialysis session**			
VLF-1	4.76 (4.12–5.98)	5.24 (4.10–6.04)	0.858
TP-1	5.58 (4.69–6.49)	5.69 (4.53–6.60)	0.973
Variance-1	5.71 (4.87–6.65)	5.71 (4.55–6.73)	0.954
nLF-1	47.20 (32.10–66.60)	44.70 (26.30–61.70)	0.338
nHF-1	30.30 (19.40–43.10)	29.40 (21.30–41.90)	0.908
LF/HF-1	0.41 (0.01–1.15)	0.33 (0.01–0.99)	0.671
**Middle phase of the index hemodialysis session**			
VLF-2	5.47 (4.29–6.22)	5.29 (4.25–6.16)	0.722
TP-2	5.86 (5.17–6.94)	5.94 (4.80–7.28)	0.991
Variance-2	5.99 (5.05–6.97)	5.96 (4.98–7.31)	0.794
nLF-2	48.40 (32.70–66.70)	46.70 (30.50–60.20)	0.448
nHF-2	28.00 (19.40–39.10)	29.00 (19.20–36.50)	0.982
LF/HF-2	0.47 (0.01–1.15)	0.44 (0.01–1.04)	0.901
**Late phases of the index HD session**^**+**^			
VLF-3	5.33 (3.94–6.47)	4.94 (3.96–6.17)	0.522
TP-3	5.83 (4.79–7.19)	5.67 (4.61–7.19)	0.580
Variance-3	6.05 (4.92–7.08)	5.75 (4.76–7.16)	0.558
nLF-3	47.85 (31.05–67.90)	44.65 (277.78–63.80)	0.750
nHF-3	25.00 (18.88–38.43)	28.00 (18.95–38.20)	0.837
LF/HF-3	0.52 (0.01–1.25)	0.50 (0.01–1.14)	0.735

Notes: Values are presented as median (interquartile range). P-value was calculated using an independent Student’s *t*-test. HRV-0, -1, -2, and -3 were HRV measured before hemodialysis, and at initial, middle, and late phases of the index hemodialysis session, respectively.

Units: Ln (ms^2^) in VLF, TP, and Variance; Ln (ratio) in LF/HF ratio; normalized unit in nLF and nHF.

Abbreviations: Ln, nature logarithmical; nHF, normalized high-frequency; nLF, normalized low-frequency; TP, total power; Variance, the variance of the R-R intervals; VLF, very-low-frequency.

**Figure 2:**
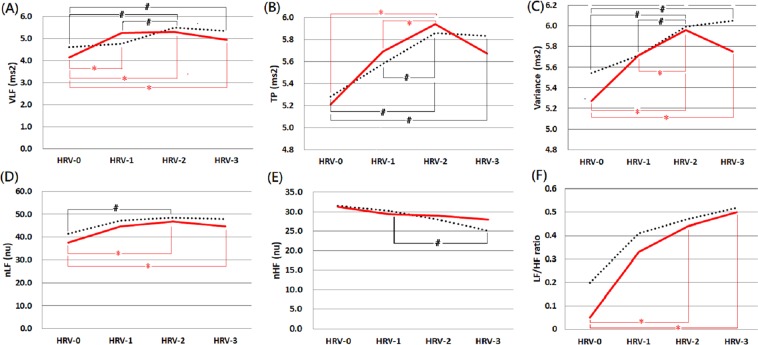
Comparisons of heart rate variability indices between survivors and non-survivors within 8-years follow-up period. Notes: These HRV indices included (**A**) VLF, (**B**) TP, (**C**) Variance, (**D**) nLF, (**E**) nHF and (**F**) LF/HF. The solid red line denotes non-survivors, while the dotted black line denotes survivors. HRV-0, -1, -2, and -3 were HRV measured at baseline, along with initial, middle, and late phases of the index hemodialysis session, respectively. No statistical difference between the two groups at any time points. ^#^ and * denote statistically different values (p ≦ 0.05) between two time-points of survivors and non-survivors, respectively. Abbreviations: nHF, normalized high-frequency; nLF, normalized low-frequency; Variance, the variance of the R-R intervals; VLF, very-low-frequency.

As to the only one exception, nHF, it exhibited a decreasing trend during the process of hemodialysis in both groups. Moreover, the values at the end of the index hemodialysis were higher in non-survivors than survivors (Fig. 2).

### Independent predictors for cardiovascular mortality at 8-year follow-up

After putting all the baseline and clinical variables in Table 1 into the multivariate Cox regression model, we found that none of them was demonstrated as an independent predictor for 8-year cardiovascular mortality. Then we put all the HRV indices, which were repeated measured into the joint modeling method to determine the independent predictors for cardiovascular mortality. Finally we found that increased nHF values (hazard ratio [HR] 1.033, 95% confidence interval [CI] 1.029–1.036, P < 0.001) during the hemodialysis process was an independent predictor for 8-year cardiovascular mortality. Oppositely, increased levels of VLF (HR 0.990, 95% CI 0.986–0.993, P < 0.001), Variance (HR 0.991, 95% CI 0.987–0.994, P < 0.001), nLF (HR 0.999, 95% CI 0.999–1.000, P = 0.006), and LF/HF ratio (HR 0.796, 95% CI 0.746–0.849, P < 0.001) during the hemodialysis process were independently protected the patients from subsequent cardiovascular mortality (Table 3).

**Table 3:** Independent predictors for cardiovascular mortality after an 8-year follow-up.

Variables	B	HR	95% CI	p-value
**Baseline characteristics and procedures**				
Nil	—	**—**	**—**	**—**
**HRV indices**				
VLF^a^	−0.011	0.990	0.986–0.993	<0.001
TP^a^	−0.001	0.999	0.998–1.001	0.321
Variance^a^	−0.010	0.991	0.987–0.994	<0.001
nLF^a^	0.000	0.999	0.999–1.000	0.006
nHF^a^	0.032	1.033	1.029–1.036	<0.001
LF/HF^a^	−0.228	0.796	0.746–0.849	0.012

Note: All HRV measurements had been adjusted by mixed models and subsequently put into a multivariate Cox regression method with adjustment to baseline characteristics and clinical variables. In the mixed model, VLF, TP, and nLF were adjusted by times (HRV-0, -1, -2, -3); Variance was adjusted by times (HRV-0, -1, -2, -3) and comorbidities with peripheral arterial disease; nHF was adjusted by times (HRV-0, -1, -2, -3) and age; LF/HF was adjusted by times (HRV-0, -1, -2, -3), age, and blood urea nitrogen. ^a^Every increment of one unit.

Units: Ln (ms2) in VLF, TP, and variance; Ln (ratio) in LF/HF ratio; normalized units in nLF and nHF.

Abbreviations: B, beta coefficient; CI, confidence interval; HR, hazard ratio; HRV, heart rate variability; Ln, nature logarithmical; nHF, normalized high-frequency; nLF, normalized low-frequency; TP, total power; Variance, the variance of the R-R intervals; VLF, very-low-frequency.

### Independent predictors for infection-associated mortality at 8-year follow-up

Multivariate Cox proportional hazards model disclosed that higher age (HR 19.29, 95% CI 3.76–99.03, P < 0.001) and lower serum albumin (HR 0.01, 95% CI 0.00–0.17, P = 0.001) predicted infection-associated mortality. When joining repeatedly measured HRV indices into the above-mentioned independent risk factors, increased nHF values (HR 1.033, 95% CI 1.029–1.036, P < 0.001) during the hemodialysis process was also found as an independent predictor for infection-associated mortality (Table 4).

**Table 4:** Independent predictors for infection-associated mortality after an 8-year follow-up.

Variables	B	HR	95% CI	p-value
**Baseline characteristics and procedures**				
Age^a^	2.96	19.29	3.76–99.03	<0.001
Albumin^a^	−4.32	0.01	0.00–0.17	0.001
**HRV indices**				
VLF^a^	0.001	1.001	0.997–1.005	0.597
TP^a^	0.001	1.000	0.999–1.001	0.354
Variance^a^	0.001	1.001	1.000–1.001	0.204
nLF^a^	0.001	1.000	1.000–1.001	0.056
nHF^a^	0.013	1.013	1.009–1.017	<0.001
LF/HF^a^	0.018	1.018	0.995–1.041	0.128

Note: All HRV measurements had been adjusted by mixed models and subsequently put into a multivariate Cox regression method with adjustment to baseline characteristics and clinical variables. In the mixed model, VLF, TP, and nLF were adjusted by times (HRV-0, -1, -2, -3); Variance was adjusted by times (HRV-0, -1, -2, -3) and comorbidities with peripheral arterial disease; nHF was adjusted by times (HRV-0, -1, -2, -3) and age; LF/HF was adjusted by times (HRV-0, -1, -2, -3), age, and blood urea nitrogen. ^a^Every increment of one unit.

Units: Ln (ms^2^) in VLF, TP, and variance; Ln (ratio) in LF/HF ratio; normalized units in nLF and nHF.

Abbreviations: B, beta coefficient; CI, confidence interval; HR, hazard ratio; HRV, heart rate variability; Ln, nature logarithmical; nHF, normalized high-frequency; nLF, normalized low-frequency; TP, total power; Variance, the variance of the R-R intervals; VLF, very-low-frequency.

### Influences of HRV indices on mortality at varied follow-up period

To confirm the influences of HRV indices on both cardiovascular and infection- associated mortality, we additionally performed the survival analyses at 4-year and 6-year follow-up. The same as the survival analysis at 8-year follow-up, all HRV measurements had been adjusted by mixed models and subsequently put into the multivariate Cox regression method with adjustment to baseline characteristics and clinical variables.

For cardiovascular mortality, the influences of the HRV indices were very consistent. The independent impacts of LF/HF and nLF on cardiovascular mortality persisted since 4-year follow-up to 8-year follow-up, whereas the independent influence of VLF and nHF were noticed at 6-year and 8-year follow-up (Table S2). As for infection-associated mortality, increased nHF oppositely played a protective role against mortality at 4-year follow-up (Table S3).

## Discussion

After an eight-year follow-up, the current study demonstrated some HRV indices as independent predictors for cardiovascular mortality and infection-associated mortality. There were several distinguishing features in the current study compared with previous reports evaluating the association between HRV and patients’ prognoses. First, we used a joint modeling method that could calculate the effects of the four repeated measurements of HRV indices and provided more value in the statistical results. To the best of our knowledge, the joint model has rarely been applied in previous HRV studies. Most previous studies used traditional models such as the linear mixed model for longitudinal data and the Cox proportional hazards model for time-to-event data. However, the two methods do not expect dependencies between these two different data types. Joint models bring longitudinal and time-to-event data simultaneously into a single model, which can assume the association between the longitudinal data and time to the event. Besides, joint models also could analyze repeated measurements and survival data synchronously, which reduce bias and provide improvements in efficiency in the assessment of prognostic factors^22^. Second, the independent association between HRV indices and infection-associated mortality shown in the current study was rarely reported previously. Third, the clinical relevance of nLF and nHF for patients’ prognoses shown in the current study was less evaluated previously. Indeed, LF and HF have been reported as prognostic predictors. However, when the spectral components are presented with absolute units (ms^2^), the changes in total power influence LF and HF simultaneously and do not reflect the real significance of LF and HF. Thus nLF and nHF were more relevant than LF and HF, respectively^23–25^. Fourth, the participant number in the current study was sufficiently large, and the eight-year follow-up period was relatively long comparing to most of the previous studies.

In the whole cohort, most of the HRV indices (except nHF) tended to increase initially in response to the stress caused by hemodialysis, but a decrease in values or the increasing tendency subsequently when the stress increased gradually. Moreover, the tendency of “decreasing HRV” in the late phase of hemodialysis was more significant in non-survivors than in survivors. Besides, the survivors seemed to have higher mean values of these HRV indices (except nHF) at most of the measurements, although the difference did not reach statistically significant (Fig. 2).

In the current study, lower variance, nLF, LF/HF ratio, and VLF, as well as higher nHF, were independently associated with higher cardiovascular mortality within the eight-year follow-up period. Lower variance reflects lower total power of ANS, lower nLF indicates lower activity of both the sympathetic and parasympathetic tone, while lower LF/HF ratio represents a lower sympathovagal balance. In the previous works, the lower LF/HF ratio was reported as a significant risk factor of cardiovascular disease in patients with CKD stage 3–5^9^, and an independent predictor of mortality in patients on peritoneal dialysis after adjustment to other predictors including age, urine volume, renal Kt/V and high-sensitivity C-reactive protein^26^. Besides, a low LF/HF ratio was found to be associated with intradialytic hypotension among patients on hemodialysis, which resulted in subsequent adverse outcomes^4,27–29^.

Although VLF was thought to reflect vasomotor function, the renin-angiotensin-aldosterone system, and parasympathetic systems^24,30^, the physiologic roles of VLF are relatively unclear compared to with other HRV indices. Nevertheless, reduced VLF power has been reported as a powerful predictor of ventricular tachycardia in patients with prior myocardial infarction and cardiovascular events in heart failure patients^31,32^. Moreover, low VLF was previously found to be associated with increased major adverse cardiovascular events and hospitalization in hemodialysis patients^33^, which was in line with our study results.

As to the role of nHF, the adverse role of nHF for cardiovascular mortality was opposite from the protective role from some other studies^9,12,13^, whereas the difference was considered as a matter of “timing of HRV measurement.” Different from other HRV indices that increased gradually since the initiation of hemodialysis, nHF started to decrease from the beginning of hemodialysis.

Taken all the HRV indices together, we could emphasize the importance of the ability to increase ANS, including sympathetic and parasympathetic activity in response to any stimulus^16^, whereas the increase of sympathetic activities is more significant than parasympathetic activities among both components of ANS. Since sympathetic and parasympathetic activities play a “growth and decline” fashion in ANS. The decreasing trend of nHF during hemodialysis in our participants probably reflects the increasing trend of sympathetic power. Thus the less decreased levels of nHF during the hemodialysis process in the non-survivors cough further explain the less sympathetic activation status in response to stress, which is straightforwardly an unfavored response. Besides, higher nHF denotes higher parasympathetic activities, a status more likely to develop hypotension and bradycardia, which were harmful in the critical illness. The decreased HRV during hemodialysis denotes the decreased ability for adequate compensation in response to external stress. These patients with decreased HRV might be susceptible to a worse outcome in critical circumstances.

Besides, lower serum albumin levels, along with older age and higher nHF, were independent predictors for infection-associated mortality. Hypoalbuminemia and old age were well-known risk factors for poor outcomes and were published previously^15^. As to the association between HRV indices and infection-associated mortality, a systematic review disclosed that low values of several HRV indices, including variance, TP, VLF, LF, LF/HF ratio, and nLF could predict mortality in sepsis patients^34^.

This effect of nHF on infection-associated mortality had not been summarized in the recent systemic review^34^. Although some studies suggested that vagal activities might be beneficial in sepsis^35,36^, other investigations reported that the HF was significantly higher in the nonsurvivors than in the survivors among septic patients^37,38^. The possible mechanism might be that sympathetic activity plays an essential role in maintaining blood pressure in patients with severe sepsis and high HF (represents parasympathetic activity) may attenuate this response. Our study disclosed that high nHF protected patients against infection-associated mortality at shorter-term (four years), but increased risks of infection-related deaths at longer-term (eight years). The exact reason or explanation for these findings is not known but worthy of further investigation.

Two previous works were worthy comparing with the current study. Chen *et al*.^15^ evaluated the ability of “the change in HRV before and after hemodialysis” for predicting mortality using a cohort of 182 hemodialysis patients. After a median follow-up period of 35.2 months, the authors demonstrated that lower values of “the change of nLF” was an independent predictor for both all-cause mortality and cardiovascular mortality among patients receiving hemodialysis. These findings pointed out the concept that the “dynamic change of ANS levels in response to external stress” might be more important than “a single measurement of ANS level.” However, as shown in Fig. 1, the dynamic changes of HRV indices values were more likely to be “reverse-U shape” than “linear shape.” Using HRV values measured at only two time-points (before and after hemodialysis) might probably oversimplify the physiological process of ANS and miss some essential interpretation. In the current study, HRV indices were measured four times from before hemodialysis to the late phase of the index hemodialysis. The “reverse-U shape” of HRV plots exhibited the physiological process of ANS in response to stress, and our study confirmed the predictive role of HRV for patients’ prognoses.

More recently, Kuo *et al*.^14^ evaluated the association between a single measurement of HRV and long-term survival (12 years) using a prospective cohort containing 41 patients. After a median follow-up period of 150.2 months, a high LF/HF ratio measured before hemodialysis initiation was found as an independent predictor for all-cause mortality (HR 3.298, P = 0.029), but not for cardiovascular mortality. The role of the LF/HF ratio disclosed in the work of Kuo *et al*.^14^ was not concordant with the results of our current study and other previous investigations in which a higher LF/HF ratio represents a better total ANS activity and better patient prognosis^9,26^. Among the possible explanations for the discordant findings, “small sample size” is an essential reason. The enrolled number of 41 was too small to make the statistical analysis meaningful. That is probably also the reason that some well-known risk factors, such as old age and low serum albumin level, did not show their adverse effect in the study of Kuo *et al*.^14^.

Several limitations need to be addressed. First, we did not use time-domain HRV indices, e.g., the SDNN, which might provide more information on autonomic nervous systems, although Variance may reflect the physiological significance of SDNN. Second, the HRV indices were only measured for four times before and during one hemodialysis session. Third, the sympathetic tone in participants was not estimated by direct methods such as muscle sympathetic nerve activity or plasma catecholamines levels, which may be able to verify the activities of the sympathetic nervous system. However, these direct methods are invasive and less clinically utility, and their predictive values have yet to be established^39^.

## Conclusions

In conclusion, abnormal function of ANS is associated with increased mortality, which is related to cardiovascular and infection origin. HRV measurement, by reflecting the various aspects of ANS activities, is a simple and useful tool to predict long-term mortality among hemodialysis patients.

## Supplementary information

Supplementary information.
