# Supplementary material for: Heart rate variability as an independent predictor for 8-year mortality among chronic hemodialysis patients
Source: Sci Rep. 2020 Jan 21;10:881. doi: 10.1038/s41598-020-57792-3 (PMC6972735; doi:10.1038/s41598-020-57792-3)
Supplement: Supplementary file 1 — Supplementary information. [file 41598_2020_57792_MOESM1_ESM.docx]

**Heart rate variability as an independent predictor for 8-year mortality among chronic hemodialysis patients**

Yu-Ming Chang, MD^1,#^, Ya-Ting Huang, RN, MN, PhD^2,#^, I-Ling Chen, RN^2^, Chuan-Lan Yang, RN^2^, Show-Chin Leu, RN^2^, Hung-Li Su, RN^2^, Jsun-Liang Kao, MD^1^, Shih-Ching Tsai, MD^1^, Rong-Na Jhen, MD^1^, Chih-Chung Shiao, MD^1,3*^.

**Supplementary information**

**Table S1. Collinearity Statistics of the variables**

| **Variables** | **Tolerance** | **Variance inflation factor** |
| --- | --- | --- |
| **Age, years** | 0.384 | 2.606 |
| **Body mass index, kg/m^2^** | 0.615 | 1.625 |
| **Gender** | 0.358 | 2.796 |
| **Causes of uremia** | 0.315 | 3.172 |
| **Period of dialysis, years** | 0.315 | 3.172 |
| **Diabetes mellitus** | 0.338 | 2.958 |
| **Hypertension** | 0.369 | 2.711 |
| **Liver cirrhosis** | 0.668 | 1.497 |
| **Coronary artery disease** | 0.534 | 1.872 |
| **Heart failure** | 0.668 | 1.498 |
| **Cerebrovascular accident** | 0.566 | 1.767 |
| **Peripheral arterial disease** | 0.467 | 2.144 |
| **Chronic obstructive pulmonary disease** | 0.493 | 2.029 |
| **Malignancy** | 0.552 | 1.813 |
| **Cardio-Thoracic Ratio, %** | 0.415 | 2.408 |
| **Actual ultrafiltration, kg** | 0.442 | 2.262 |
| **Blood urea nitrogen, mg/dL** | 0.456 | 2.193 |
| **Creatinine, mg/dL** | 0.561 | 1.783 |
| **Kt/V** | 0.253 | 3.960 |
| **Calcium, mg/dL** | 0.419 | 2.386 |
| **Phosphate, mg/dL** | 0.374 | 2.671 |
| **Albumin, g/dL** | 0.452 | 2.213 |
| **Sodium, mmol/L** | 0.459 | 2.178 |
| **Potassium, mEq/L** | 0.515 | 1.943 |
| **Intact-parathyroid hormone, ug/L** | 0.342 | 2.922 |
| **Hemoglobin, g/dL** | 0.468 | 2.135 |
| **White blood cell, x10^9^/L** | 0.343 | 2.915 |
| **Triglyceride, mg/dL** | 0.546 | 1.831 |
| **Sugar (non-fasting), mg/dL** | 0.341 | 2.929 |

**Note:** tolerance > 0.1 or variance inflation factor < 10 denote that the variable pass the collinearity test.

**Table S2. Independent predictors for cardiovascular mortality after various periods of follow-up**

| **HRV indices** | **4-year follow-up**  **(5 patients died)** | **6-year follow-up**  **(11 patients died)** | **8-year follow-up**  **(13 patients died)** |
| --- | --- | --- | --- |
| VLF ^a^ | 1.000 (0.997 - 1.003) | 0.988 (0.982 - 0.993)*** | 0.990 (0.986 - 0.993)*** |
| TP ^a^ | 1.000 (0.998 - 1.002) | 1.000 (0.999 - 1.001) | 0.999 (0.998 - 1.001) |
| Variance ^a^ | 1.000 (0.997 - 1.002) | 1.000 (0.999 - 1.001) | 0.991 (0.987 - 0.994)*** |
| nLF ^a^ | 0.996 (0.996 - 0.997)*** | 0.998 (0.996 - 0.999)* | 0.999 (0.999 - 1.000)** |
| nHF ^a^ | 1.010 (0.999 - 1.02) | 1.005 (1.001 - 1.008)** | 1.033 (1.029 - 1.036)*** |
| LF/HF ^a^ | 0.517 (0.516 - 0.517)*** | 0.685 (0.685 - 0.685)*** | 0.796 (0.746 - 0.849)* |

**Notes:** Values are presented as hazard ratio (95% confidence interval).

All HRV measurements had been adjusted by mixed models and subsequently put into a multivariate Cox regression method with adjustment to baseline characteristics and clinical variables. In the mixed model, VLF, TP, and nLF were adjusted by times (HRV-0, -1, -2, -3); Variance was adjusted by times (HRV-0, -1, -2, -3) and comorbidities with peripheral arterial disease; nHF was adjusted by times (HRV-0, -1, -2, -3) and age; LF/HF was adjusted by times (HRV-0, -1, -2, -3), age, and blood urea nitrogen. ^a^ every increment of one unit;

*, **, *** denote p <0.05, 0.01, 0.001, respectively.

**Abbreviations:** LF/HF, low-frequency/high-frequency ratio; HRV, heart rate variability; nHF, normalized high-frequency; nLF, normalized low-frequency; TP, total power; Variance, the variance of the R-R intervals; VLF, very-low-frequency.

**Table S3. Independent predictors for infection-associated mortality after various periods of follow-up**

| **HRV indices** | **4-year follow-up**  **(32 patients died)** | **6-year follow-up**  **(40 patients died)** | **8-year follow-up**  **(49 patients died)** |
| --- | --- | --- | --- |
| VLF ^a^ | 0.999 (0.995 - 1.003) | 0.999 (0.998 - 1.001) | 1.001 (0.997 - 1.005) |
| TP ^a^ | 1.000 (0.999 - 1.001) | 1.000 (0.999 - 1.001) | 1.000 (0.999 - 1.001) |
| Variance ^a^ | 1.000 (0.999 - 1.001) | 0.999 (0.997 - 1.001) | 1.001 (1.000 - 1.001) |
| nLF ^a^ | 0.999 (0.999 – 1.000) | 1.000 (0.999 - 1.000) | 1.000 (1.000 - 1.001) |
| nHF ^a^ | 0.997 (0.996 - 0.998)*** | 1.000 (0.998 - 1.003) | 1.013 (1.009 - 1.017) ** |
| LF/HF ^a^ | 1.079 (0.775 - 1.501) | 1.030 (0.617 - 1.721) | 1.018 (0.995 - 1.041) |

**Notes:** Values are presented as hazard ratio (95% confidence interval).

All HRV measurements had been adjusted by mixed models and subsequently put into a multivariate Cox regression method with adjustment to baseline characteristics and clinical variables. In the mixed model, VLF, TP, and nLF were adjusted by times (HRV-0, -1, -2, -3); Variance was adjusted by times (HRV-0, -1, -2, -3) and comorbidities with peripheral arterial disease; nHF was adjusted by times (HRV-0, -1, -2, -3) and age; LF/HF was adjusted by times (HRV-0, -1, -2, -3), age, and blood urea nitrogen. ^a^ every increment of one unit;

*, **, *** denote p <0.05, 0.01, 0.001, respectively.

**Abbreviations:** LF/HF, low-frequency/high-frequency ratio; HRV, heart rate variability; nHF, normalized high-frequency; nLF, normalized low-frequency; TP, total power; Variance, the variance of the R-R intervals; VLF, very-low-frequency.
